# The COVID-19 pandemic and the timing of government response: A comparison of four Nordic countries March–June 2020

**DOI:** 10.1177/14034948231171201

**Published:** 2023-04-28

**Authors:** Martin Lindström

**Affiliations:** Social Medicine and Health Policy, Department of Clinical Sciences and Centre for Primary Health Care Research, Lund University, Sweden

**Keywords:** Pandemic, coronavirus, COVID-19 mortality, Swedish strategy, government response, restrictions, social distancing, Sweden

## Abstract

**Objectives:**

The aim is to compare Sweden, Denmark, Finland and Norway regarding government response to the COVID-19 pandemic in mid-March–June 2020 using the Oxford Government Response Tracker.

**Study design:**

Descriptive longitudinal ecological study.

**Methods:**

Descriptive analysis of time series data.

**Results:**

Sweden displayed a far lower response index in March. By late April indexes were similar. In May–June, response indexes were lower in Finland and Norway than in Sweden. The average response index in mid-March–June was similar in Sweden, Finland and Norway.

**Conclusions:**

**The government response in the four countries indicates that timing of response was essential. Sweden’s slow and weak initial government response in March–April was followed by less loosening of government response in May–June compared with, especially, Finland and Norway, which resulted in similar average government response in mid-March–June for the three countries. As a comparison, COVID-19 mortality per capita was 10 times higher in Sweden than in Finland and Norway, and five times higher than in Denmark during the same period.**

## Introduction

The COVID-19 outbreak unfolded rapidly in early 2020. On 11 March, the World Health Organization (WHO) proclaimed it a pandemic. Very limited evidence existed regarding the duration of immunity following infection [1]. Intervention and longitudinal observation studies were not available. Many European countries, including Denmark, Finland and Norway, aimed to eradicate the contagion following WHO recommendations regarding social distancing, testing and contact tracing [2], although the measures were not identical.

The official Swedish strategy aimed to reduce the spread of the coronavirus to limit the strain on the healthcare system and protect vulnerable groups such as the elderly and other risk groups. It also included aims to reduce the impact on important functions in society, reduce consequences for the public and companies, reduce anxiety and implement the right measures at the right time [3].

Restrictions in Sweden in March and early April were initially considerably looser than in the other Nordic countries, but just weeks later these differences between the Nordic countries became smaller. The Swedish Public Health Agency **(***Folkhälsomyndigheten* (FHM)) recommended to all regions in mid-March 2020 that testing should be restricted to in-hospital patients and risk groups [4]. This policy was in effect until well into May 2020. Many measures, such as lowering the number of participants allowed in public gatherings, restrictions of entry into the country, restrictions regarding visits to care homes for the elderly, were considerably fewer, slower and weaker than in the other Nordic countries [5].

The initial Swedish strategy implemented by the FHM seemed remarkable given the historical evidence from communicable diseases prevention as well as previous historical pandemics. International public health cooperation started with the International Sanitary Conference in Paris in 1851 to restrict the spread of communicable infectious diseases. A total of 14 such international conferences were held in 1851–1938, followed after the Second World War by the founding of the WHO in 1948 and the International Sanitary Regulations (ISRs) issued in 1951 by the WHO. The ISRs were later followed by the International Health Regulations in 1969, which broadened the perspective to non-communicable diseases and the broader concept of international health [6]. Later, international health was further developed, most importantly by the Alma-Ata Declaration (1978) into the concept of global health, further incorporating equality, socioeconomic differences, the role of public institutions and organizations and the incorporation of primary healthcare into healthcare systems [7]. It should still be remembered that international cooperation started with prevention of communicable diseases. In contrast, the leading mastermind behind the initial Swedish strategy exclaimed already in early April 2020 that ‘All other countries are wrong’, disregarding international cooperation [8].

Comparatively recent historical work also indicates that early and sustained implementation of restrictions on varying aspects of social contacts significantly reduced mortality rates during the 1918–1919 influenza pandemic [9]. In a study of 23 cities in the USA a strong correlation was found between excess mortality and the lack of early timing of interventions [10]. This experience was not acknowledged, probably partly because the FHM at the time of its foundation on 1 January 2014 had been disconnected from the direct access to university-co-employed professors/researchers also conducting active research within the authority that had characterized its predecessors *Statens Bakteriologiska Laboratorium* (1937–1993) and *Statens Smittskyddsinstitut* (1993–2014) [11].

Given the high death tolls from COVID-19 in Sweden compared with the other Nordic countries, the opposition parties insisted that a Corona commission should scrutinize the Swedish strategy and report its results well before the 2022 parliamentary election. On 15 December 2020, the first Corona commission report concerning the care of the elderly during the pandemic concluded that the Swedish strategy had failed to protect the elderly and that the main reason was the high general spread in the population. There had also been a lack of protection equipment, and problems with organization and staffing [12]. The second Corona commission report, published on 29 October 2021, and the final commission report, published on 25 February 2022, concluded that the FHM and the government had implemented too few, too late and too weak measures that resulted in unacceptably high spread of the coronavirus. Returning travellers from winter sports vacation abroad should have been placed in quarantine, travel into Sweden should have been prohibited earlier and the allowed number of participants in public events should have been restricted much more and at an earlier point in time. There should have been a law supporting closure of public places, shopping centres, public indoor swimming pools, hair salons and cultural and sports events. Facemasks should have been recommended by the FHM following evidence. The same government authority (FHM) should not handle both public health and protection against infectious diseases, the government should have consulted other academic voices and authorities than the FHM and considered the measures taken by other governments, particularly in the other Nordic countries [13]. Given these conclusions by the Corona commission, it would be relevant to quantify government response during the first wave of the pandemic from mid-March to June 2020. The Oxford Government Response Tracker presents an opportunity for such a descriptive quantification of ecological-level data.

The aim of this study is to compare Sweden, Denmark, Finland and Norway regarding government response using the Oxford Government Response Tracker index for the spring of 2020.

## Methods

This short communication utilizes descriptive ecological national-level data from the Oxford University Government Response Tracker. A comparison of response indexes was presented for Sweden, Denmark, Finland and Norway for the period mid-March until end of June 2020. The government response tracker index measure ranges from 0 (no government response) to 100 (theoretically complete government response). The index includes school closing, workplace closing, cancelling of public events, restrictions on gatherings, closing of public transport, stay at home requirements, restrictions on internal movement, international travel controls, income support for households, debt/contract relief, fiscal measures, economic support to other countries, public information campaigns, testing policy, contact tracing, emergency investment in healthcare, investment in vaccines, facial coverings, vaccination policy, and protection of elderly people for the study period [14].

The government response indexes for the mid-March–June 2020 period are displayed in Table I.

**Table I.** Oxford Government Response Tracker indexes for Sweden, Denmark, Finland and Norway at two-week intervals from mid-March until the end of June 2020.

	Sweden	Denmark	Finland	Norway
16 March 2020	22.20	65.74	57.41	60.19
31 March 2020	53.70	72.22	67.59	79.63
15 April 2020	64.81	68.52	62.96	79.63
30 April 2020	64.81	68.52	60.19	67.59
16 May 2020	64.81	68.52	53.70	58.33
31 May 2020	64.81	60.19	48.15	58.33
15 June 2020	59.26	57.41	35.19	40.74
30 June 2020	59.26	57.41	35.19	40.74
Average of eight dates from mid-March until end of June 2020	56.71	64.82	52.55	60.65

Sources:

Phillips T. *Codebook for Oxford COVID-19 Government Response Tracker*. Oxford: Oxford University, 24 May 2021.

Hale T, Angrist N, Goldszmidt R, et al. A global panel database of pandemic policies (Oxford COVID-19 Government Response Tracker). *Nat Hum Behav* 2021, https://doi.org/10.1038/s41562-021-01079-8

## Results

Table I shows that on 16 March 2020, the Oxford COVID-19 Response Tracker index was 22.20 in Sweden, 65.74 in Denmark, 57.41 in Finland and 60.19 in Norway. On 31 March, Sweden still had a lower 53.70 index than 72.22 for Denmark, 67.59 for Finland and 79.63 for Norway, respectively. In mid-April, Sweden had increased its government response index further to 64.81, which was in fact higher than the 62.96 index value for Finland, while Denmark’s index had increased to 68.52 and Norway’s 79.63 index was completely unchanged. Sweden’s index then remained unchanged at 64.81 from mid-April until the end of May, while Finland and Norway successively started to loosen government response from mid-April to finally reach indexes 48.15 and 58.33 at the end of May, respectively. Denmark’s government response decreased to 60.19 at the end of May. In June, Sweden’s index was a notably higher 59.26 compared with Finland’s 35.19 and Norway’s 40.74, and somewhat higher than Denmark’s 57.41.

The government response index averages over the mid-March until late June period are closely similar 56.71 for Sweden, 52.55 for Finland and 60.65 for Norway, while Denmark’s average was 64.82.

## Discussion

The Swedish strategy was characterized by a slower and weaker initial response in mid-March–April, but over the entire mid-March until June period the response index was on average similar for Sweden, Finland and Norway, and somewhat higher for Denmark.

In Denmark, Finland and Norway, the initial response was directed by the government. In Sweden, the response was directed by the state authority FHM in the spring, in essence trusted by the government to handle the pandemic.

The weak Swedish government response in March and early April 2020 may have resulted in a situation in which it was hard to lift government response measures in late spring/early summer due to the high spread and the high death toll. The sixth aim of the official Swedish strategy to ‘implement the right measures at the right time’ [3] seems to have failed with regard to timing in the spring of 2020. The conclusion is that timing of government response seems to be essential.

The Oxford Government Response Tracker index measures combined government response at different levels of government. The decisions and measures made by the regions responsible for the healthcare system and the municipalities responsible for the care homes for the elderly in Sweden are also a part of government response in the Oxford index, but the entire Swedish government and public response was directed by the FHM in the spring of 2020.

The project assessing the Government Response Tracker index included more than 400 reporters (mostly at postdoc level) from different countries who underwent a training programme to learn to gather policy data from public sources and validated sources in the mass media. In the next step, there was also a central validation team that checked missing data and validity of reported data (see Hale et al. [14]). For reasons of historical, cultural, social and political similarities, comparisons between the Nordic countries would most probably be more valid and prone to less error than other international comparisons. Internationally, poorer countries display substantially weaker associations between the implementation of stricter policies and COVID-19 mortality, while on the other hand stronger positive associations between stricter policies and lower COVID-19 mortality were observed in high-income countries [15]. A further strength is that the response data were collected completely independently of the research question. The risk of ecological fallacy is negligible given that the main government response index data measures ecological-level policies and not ecological-level aggregates of individual-level data.

The total COVID-19 death toll reported to the WHO during the period mid-March until end of June 2020 was 5310 in Sweden, 605 in Denmark, 328 in Finland and 249 in Norway until 30 June 2020 [16]. The per capita death tolls were approximately 10 times higher for Sweden (51.16/100,000) than Finland (6.15/100,000) and Norway (4.61/100,000) and five times higher than Denmark (10.36/100,000). Internationally, potential under-reporting of COVID-19 mortality has been measured as excess (above expected) mortality compared with registered COVID-19 mortality. Such estimations for the years 2020–2021 indicate that excess mortality compared with COVID-19 mortality was relatively lowest in Norway, relatively higher in Sweden, still relatively higher in Denmark and highest relative to total excess mortality in Finland. However, all Nordic countries strongly differed from countries, in particular Africa and most of Asia, where under-reporting was relatively much more common [17].

This short communication concerns only the initial Swedish strategy during the first pandemic wave from mid-March to June 2020, which displayed very high Swedish COVID-19 mortality in the European context. In the longer perspective, Swedish COVID-19 mortality progressed in relative terms from high levels to approximately average European mortality for several reasons not possible to fully disentangle in a short communication. Plausible explanations include alterations of the strategy, including harsher restrictions imposed by the Swedish government in late 2020 following the outbreak of the second wave also in Sweden (which was contrary to expectations motivated by the expected achievement of close to herd immunity following the initial strategy), the start of vaccinations around New Year 2020/2021 and the probable influence of autocorrelation. If many frail individuals died in Sweden due to higher spread of the virus compared with the neighbouring countries, then mortality might become relatively lower in 2021 due to the relative absence of medically frail individuals. In addition, it should also be noted that Sweden for a long time has been one of the western countries with fewest hospital care places per capita [18,19], which may also have contributed to the high COVID-19 mortality in Sweden. Furthermore, waiting times to planned healthcare are also higher in Sweden than in many other countries and patients with chronic diseases are treated adequately to a lower extent [20]. In Region Stockholm, the most populous of all 21 Swedish regions responsible for healthcare and most affected by the pandemic in the spring of 2020, many elderly inhabitants were denied adequate healthcare such as oxygen and hydration in the first pandemic wave in the spring of 2020 [21].

In contrast to COVID-19 mortality comparisons, COVID-19 incidence comparisons between the Nordic countries during the March–June 2020 period would probably be impossible, because Sweden deliberately stopped testing and contact tracing from mid-March with only in-hospital patients and particularly vulnerable risk groups as exceptions. This policy was maintained almost until mid-May, when testing was gradually resumed.

## Conclusions

The government response in the four countries indicates that timing of response was essential. Sweden’s slow and weak initial government response in March–April was followed by less loosening of government response in May–June compared with, especially, Finland and Norway, which resulted in similar average government response in mid-March–June for the three countries. As a comparison, COVID-19 mortality per capita was 10 times higher in Sweden than in Finland and Norway, and five times higher than in Denmark during the same period.

## References

[1] Kissler SM Tedijanto C Goldstein E , et al. Projecting the transmission dynamics of SARS-CoV-2 through the postpandemic period. Science 2020;14:1-17. 10.1126/science.abb5793 PMC7164482 32291278

[2] Lindström M. The COVID-19 pandemic and the Swedish strategy: Epidemiology and postmodernism. SSM Popul Health 2020;11:100643. 32885019 10.1016/j.ssmph.2020.100643 PMC7453138

[3] Regeringskansliet. Regeringens arbete med coronapandemin. Strategi med anledning av det nya coronaviruset. Stockholm: Regeringskansliet, 7 April 2020, https://www.regeringen.se/regeringens-politik/regeringens-arbete-med-coronapandemin/strategi-med-anledning-av-det-nya-coronaviruset/ (2020, accessed 26 February 2022).

[4] Karlsson P. Nya strategin- slutar räkna exakta antalet coronafall. Stockholm: Aftonbladet, 13 March 2020 (updated 17 March), https://www.aftonbladet.se/nyheter/samhalle/a/70GQRV/nya-strategin–slutar-rakna-exakta-antalet-coronafall (2020, accessed 6 March 2021).

[5] Obminska A. “Det här vet vi om coronaviruset”. Stockholm: Ny Teknik, 2020, continually updated, https://www.nyteknik.se/samhalle/det-har-vet-vi-om-coronaviruset-6985117 (2020, accessed 31 March 2021).

[6] Birn AE. The stages of international (global) health: Histories of success or success of history? Glob Public Health 2009;4:50-68. 19153930 10.1080/17441690802017797

[7] Holst J. Global health – emergence, hegemonic trends and biomedical reductionism. Global Health 2020;16:42. 32375801 10.1186/s12992-020-00573-4 PMC7201392

[8] Grundberg Wolodarski K . “Johan Giesecke: Alla andra länder gör fel”, Dagens Industri, Stockholm, 2 April 2020, https://weekend.di.se/intervjuer/alla-andra-lander-gor-felPublic (2020, accessed 7 May 2022).

[9] Tomes N. “Destroyer and teacher”: Managing the masses during the 1918–1919 influenza pandemic. Public Health Rep 2010;125(Suppl. 3):48-62. 20568568 10.1177/00333549101250S308 PMC2862334

[10] Bootsma MCJ Ferguson NM . The effect of public health measures on the 1918 influenza pandemic in U.S. cities. Proc Natl Acad Sci U S A 2007;104:7588-93. 10.1073/pnas.0611071104 PMC1849868 17416677

[11] Vahlne A. On the virology of SARS-CoV-2 and an expert authority without real experts: Was there a deliberate disinformation from the Public Health Agency of Sweden on the SARS-CoV-2 infection’s spread in the population? In: Bergmann S Lindström M (eds) Sweden’s pandemic experience. London, New York: Routledge, 2022, pp.75-89.

[12] Melin M Ahlbäck Öberg S Enander A , et al. Coronakommissionen. Äldreomsorgen under pandemin. Stockholm: Delbetänkande av Coronakommissionen, Statens Offentliga utredningar, SOU 2020: 80, https://coronakommissionen.com/wp-content/uploads/2020/12/sou_2020_80_aldreomsorgen-under-pandemin_webb.pdf (2020, accessed 23 January 2021).

[13] Melin M Ahlbäck Öberg S Enander A , et al. Coronakommissionen. Sverige under pandemin. Vol. 1. Smittspridning och smittskydd. Stockholm: Delbetänkande av Coronakommissionen, Statens Offentliga utredningar, SOU 2021: 89, https://coronakommissionen.com/wp-content/uploads/2020/12/sou_2020_80_aldreomsorgen-under-pandemin_webb.pdf (2021, accessed 24 March 2022).

[14] Hale T Angrist N Goldszmidt R , et al. A global panel database of pandemic policies (Oxford COVID-19 Government Response Tracker). Nat Hum Behav 2021;5:529-38. 10.1038/s41562-021-01079-8 33686204

[15] Shiva M Molana H. The luxury of lockdown. Eur J Dev Res 2022;34:503-23. 10.1057/s41287-021-00389-x PMC8033558 33850346

[16] World Health Organization. Coronavirus disease 2019 (COVID-19). Situation Report-162. Geneva: World Health Organization, 2020.

[17] Wang H Paulson KR Peace SA , et al. Estimating excess mortality due to the COVID-19 pandemic: A systematic analysis of COVID-19-related mortality, 2020–2021. Lancet 2022;399:1513-36. 10.1016/S0140-6736(21)02796-3 PMC8912932 35279232

[18] Borgström A. Sverige har lägst antal vårdplatser i Europa. Läkartidningen 2007;104:396-7.

[19] Ström M. Fortsatt färre vårdplatser i Sverige. Läkartidningen 2018,115:EY66.

[20] Nilsson P. Vården ur befolkningens perspektiv. En jämförelse mellan Sverige och tio andra länder. Rapport 2021: 4. Stockholm: Myndigheten för vård- och omsorgsanalys, 2021.

[21] Zaremba M. “Varför fick de äldre dö utan läkarvård?”, Dagens Nyheter, Stockholm, 13 October 2020, https://www.dn.se/kultur/varfor-fick-de-aldre-do-utan-lakarvard/ (2020, accessed 7 May 2022).

